# Supplementary material for: Cortinarius and Tomentella Fungi Become Dominant Taxa in Taiga Soil after Fire Disturbance
Source: J Fungi (Basel). 2023 Nov 17;9(11):1113. doi: 10.3390/jof9111113 (PMC10672602; doi:10.3390/jof9111113)
Supplement: Supplementary file 1 [file jof-09-01113-s001.zip › jof-2709613-supplementary.pdf]

Table S1. Classification of fungal genera with average relative abundance>1 % into trophic modes and guilds

Trophic mode	Guild	Average number of reads			
		CK	L	M	H
Symbiotroph	Ectomycorrhizal	22.112.33±3984.73a	11879±115.94c	16.179.67±1680.25b	13.888±705.68bc
	Orchid Mycorrhizal	482±178.41c	1985.67±330.69b	2052.67±393.4b	3326.67±529.25a
Saprotroph	Wood Saprotroph	0.00	0.00	398.33±564.85a	0.67±1.15a
	Plant Saprotroph-Wood Saprotroph	81.33±38.4c	5043.67±488.64b	7241±607.48a	764±409.59c
	Undefined Saprotroph	3670.67±1837.1a	872±358.12b	872±318.06b	4465.33±728.01a
	Soil Saprotroph	965.67±484.67a	1471.33±195.54a	670.33±149.19a	1190±1280.31a
Pathotroph	Plant Pathogen	16.33±9.02a	152.33±168.35a	1096.33±1817.51a	67.67±23.46a
Pathotroph-Symbiotroph	Ericoid Mycorrhizal	414.67±120.1b	446±152.3b	739±34.77a	159±35.68c
Pathotroph-Saprotroph	Plant Pathogen-Undefined Saprotroph	208.67±256.6a	2.67±2.08a	1±1.173a	3±5.2a
Saprotroph-Symbiotroph	Undefined Saprotroph-Undefined Biotroph	2196±324.12a	0.00	0.00	0.67±1.15b
	Lichenized-Undefined Saprotroph	176±84.26b	68±19.7b	2.67±2.31b	2265.33±1162.94a
	Endophyte-Litter Saprotroph-Soil Saprotroph-Undefined Saprotroph	309.33±92.92b	511±65.09b	949.67±328.61a	582.33±40.15b
	Ectomycorrhizal-Endophyte-Ericoid Mycorrhizal-Litter Saprotroph-Orchid Mycorrhizal	18.33±20.65c	1353.67±365.47a	724±277.5b	165±59.81c
	Ectomycorrhizal-Fungal Parasite-Plant Pathogen-Wood Saprotroph	6±10.39b	501±120.2ab	34.67±9.07b	957.67±682.71a
	Dung Saprotroph-Ectomycorrhizal	13±4.36c	102.67±38.37b	361.33±64.53a	1±1.73c
Pathotroph-Saprotroph-Symbiotroph	Fungal Parasite-Undefined Saprotroph	291±179.64a	23±7b	8.33±4.04b	16.67±15.01b
	Bryophyte Parasite-Ectomycorrhizal-Ericoid Mycorrhizal-Undefined Saprotroph-Wood Saprotroph	9.67±6.11b	113±13.89b	19.67±10.79b	373±245.23a
